# Law, Ethics, Religion, and Clinical Translation in the 21st Century—A Discussion with Derek Hei

**DOI:** 10.1002/stem.323

**Published:** 2010-02-17

**Authors:** Susan Rainey Daher, Majlinda Lako, Alan O Trounson

## About Dr. Hei

Dr. Hei received his B.S. in Chemical Engineering in 1988 from the University of Wisconsin at Madison and went on to complete his Ph.D. in Biochemical Engineering at the University of California at Berkeley in 1993. He then entered industry, where he worked for seven years at Genentech and then Cerus Corp., developing recombinant protein and cell-based biotherapeutics and rising to the position of Director of Biomedical Engineering at Cerus Corp. In 2000, he joined the Waisman Center at UW–Madison to lead development of the Waisman Clinical Biomanufacturing Facility (WCBF), a non-profit technology development and clinical manufacturing support core that works with academic investigators and biotechnology companies. The WCBF recently received 8.8 million dollars in funding from the National Heart, Lung, and Blood Institute for their Production Assistance for Cell Therapeutics (PACT) program. Now Technical Director and Senior Scientist at WCBF, Dr. Hei leads a team of scientists and engineers that support academic researchers and start-up biotechnology companies that are involved in developing biotherapeutics for early-stage human clinical trials. The WCBF has advanced a variety of biotherapeutics into human clinical trials including recombinant proteins, plasmid DNA, viral vectors, and cell therapeutics. Dr. Hei has also been a Principal Investigator on the National Stem Cell Bank project. In addition to his numerous peer-reviewed publications, Dr. Hei also currently holds nine patents.

**Figure 1:**
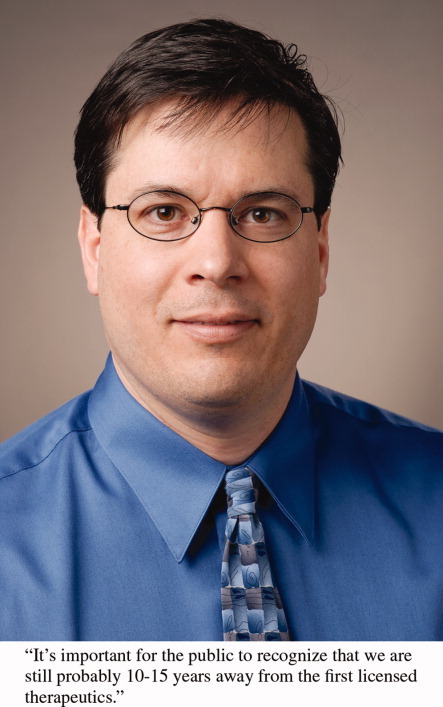
Derek Hei, PhD. University of Wisconsin Stem Cell and Regenerative Medicine Center.

## The National Stem Cell Bank and the Wisconsin International Stem Cell Bank

In September 2005, the National Stem Cell Bank (NSCB) was established in Madison, Wisconsin, as an NIH-funded resource to grow, characterize, and distribute cell lines listed on the NIH Human Pluripotent Stem Cell Registry (a list of embryonic stem cells lines eligible for use in federally funded research), and to provide comprehensive technical support to stem cell researchers around the world.

The NSCB with Derek Hei and James Thomson as Directors has been hosted by the WiCell Research Institute, a non-profit, supporting organization of the University of Wisconsin at Madison established in 1999 to focus on enhancing and expanding the study of human pluripotent stem cells by supporting basic research, establishing research protocols, and creating and distributing cell lines. They also provide training to scientists worldwide, and supporting efforts to unlock the therapeutic potential of stem cell technologies.

As Dr. Hei explains “The NSCB distributes human ES cell lines, including genetically engineered cell lines (e.g., reporter cell lines), for research applications. These cell lines are obtained through voluntary donations. The NSCB regulates the use of cells only through the Material Transfer Agreement. In signing this MTA, investigators agree that they will not perform specific types of experiments. The NSCB also requests that the investigators have their research approved by local Stem Cell Research Oversight (SCRO) committees at the researcher's institution.

Unfortunately, the NSCB's funding will end on February 28, 2010, and the NIH currently has not discussed any plans for continued funding. The Wisconsin International Stem Cell Bank (WISC) will likely continue to distribute hESC lines.” The WISC bank (http://www.wicell.org) was originally established by WiCell in 1999 to cover distribution of hES cells as well as other pluripotent stem cell lines not included in the National Stem Cell Bank. The NSCB and WiCell have shipped cells to more than 800 researchers in 32 countries and 42 states. WiCell also runs an exceptional education and outreach program that provides training for scientists and offers educational programs for K-12 students and the general community.

## “I Think That It's Important to Find Ways to Standardize Cell Characterization Methods and Establish Biomarkers of Clinical Outcomes”

The WCBF is a state-of-the-art cleanroom facility (not GMP) designed to manufacture cell and gene therapeutics for early-stage (Phase I and Phase II) human clinical trials according to the FDA's cGMP guidelines for early-stage human clinical trials. As Technical Director and Senior Scientist at WCBF, Dr. Hei interacts with academic investigators and companies to address technical issues related to the development and production of cutting-edge biotherapeutics and the standardization of results.

“For mesenchymal stem cells in particular, it seems that there are many clinical trials being conducted with varying clinical outcomes. I think that it's important to find ways to standardize cell characterization methods and establish biomarkers of clinical outcomes. This is obviously not an easy task given that we don't always clearly understand what properties are key to clinical outcomes. The development of better in vitro and in vivo potency assays could help in this regard. Genetic stability is another big issue. We need to know what key factors impact genetic stability as well as the impact of low levels of abnormal cells on the safety profile for stem cell-based therapeutics. We need to ask questions, such as whether genetic stability differs from line to line, and determine the critical steps in culture and cryopreservation that contribute to the development of abnormal karyotypes. I think that we also need a better understanding of the impact of residual undifferentiated ES cells, along with methods for detecting and removing those cells, and animal models for determining the impact of specific levels of undifferentiated cells on product safety. Geron has worked through some of these issues in preparing their data package for their Investigative New Drug (IND) filing. However, this information is confidential and is not shared with the public sector. It would be useful to have work done in this area that can be shared with other translational investigators to help investigators move through these issues to IND filings more efficiently.”

## “I Think That Clinical Development Will Be the Most Important Aspect of Stem Cell Research over the Next 10–15 Years”

“One of the most important aspects of stem cell research to be followed over the next 10 years will certainly be the development of technologies for creating iPS (induced pluripotent stem) cells. The ability to create disease models for studying developmental issues and screening potential therapeutics could be a huge near-term pay-off. The therapeutic side of iPS cells is obviously much cloudier even with the development of non-integrating reprogramming vectors. The effect of reprogramming on cell epigenetics, involvement of oncogenes in the reprogramming process, and process efficiency and reproducibility from patient to patient are some of the many key issues that need to be resolved before iPS cell-based clinical applications can become a reality.”

“That brings us to the other major development—the movement of hESC (human embryonic stem cell)-based therapeutics into the first human clinical trials. Clearly this is just the first chapter in this development, and the remaining chapters will be written in the coming years as Geron's clinical trial for spinal cord injury moves forward and others move into clinical trials—probably for eye indications. Personally, I think that clinical development will be the most important aspect of stem cell research over the next 10–15 years. However, it's important for the public to recognize that we are still probably 10–15 years away from the first licensed therapeutics.”

“It will also be important for the stem cell community to continue to work toward addressing the key technical issues that hinder successes in practical therapeutic applications since clinical applications will be the most visible sign that the battles over the ethical issues were worth fighting. Uniform regulatory requirements for therapeutic stem cells will take some time to achieve. This has been the case for other biologics that have been in development much longer than stem cells—monoclonal antibodies, for example. There are groups, such as the International Conference on Harmonisation (ICH), that work to harmonize regulations, but very often there is just general agreement at best with variations on exact requirements from country to country. I think that regulatory requirements for stem cells will continue to evolve as we learn more about potential risks through animal studies and human clinical trials. These findings will serve to shape the regulatory landscape as the field matures and the resulting regulatory requirements may differ from one country to the next.”

## “Cell Banking Is a Key Aspect of Supporting Research in This Field”

“I think that it's important to recognize the importance of cell banking, given that cell quality can have a huge impact on research quality and subsequently clinical success. Banking requires the development of an infrastructure for growing and testing hESCs. Although some labs may like the idea of receiving funding for cell banking, it is not typically a job that labs are willing to make a long-term commitment toward.” Dr. Hei notes that researchers in the United States are still working on the impact of the new NIH research guidelines that went into effect in July 2009. “Although we now have new approved hESC lines and NIH-funded hESC research projects, many of these researchers have been forced to work with new cell lines. To date, H1 is the only previous NIH Registry hESC line that has been re-approved for use in NIH-funded research. This represents a potential set-back for NIH-funded research in the U.S. since switching a research program to a new cell line is not typically a trivial matter. Cell lines can behave very differently from one another. Without a centralized source, such as the National Stem Cell Bank, for distributing cells, there may be issues with the timing for cell receipt and potentially even the quality of the cells that are distributed.”

“In my opinion, one of the biggest obstacles right now is the lack of well characterized human ES cells that are available for NIH-funded research. This can be resolved either by obtaining re-approval of the previous NIH-approved lines under the Bush administration or by establishing a centralized bank network that can help with banking, characterizing, and distributing cell lines. Another major problem is that depositing cell lines is voluntary. From my perspective, it would be more efficient to work toward a centralized banking system and encourage investigators to deposit their hESC lines.”

## “It Is Important for the Public to Understand the Practical Differences Between Human ES Cells and Adult Stem Cells”

Dr. Hei points out that although iPS cells are a promising new technology that helps to avoid many of the difficult ethical issues associated with hESCs, we are still early in the learning process. “Human ES cells will continue to be important for research and may continue to be the most suitable cell type for allogeneic cell therapy applications. iPS cells have a long way to go before there are clinical applications but may offer long-term hope to address chronic diseases where autologous therapeutics may be needed to address immune rejection issues. It is important for the public to understand the practical differences between human ES cells and adult stem cells. Education is obviously critical. These cell types are not necessarily interchangeable and each cell type may be beneficial for specific clinical applications. Unfortunately, it will likely take many years of research and human clinical studies to clearly understand the differences in therapeutic potential between hESC, iPS, and adult stem cell types.”

